# A Modified Technique of Bipolar Loop Resectoscopic Slicing for Treating Submucous Fibroids With Enucleation Makes the Operation Safer

**DOI:** 10.3389/fsurg.2021.746936

**Published:** 2021-11-10

**Authors:** Wenying Zhang, Jing Liu, Qiongwei Wu, Yu Liu, Chunchun Wang, Chengbin Ma

**Affiliations:** Department of Gynecology, Shanghai Changning Maternity and Infant Health Hospital, Shanghai, China

**Keywords:** bipolar loop, enucleation, hysteroscopy, modified technique, submucous fibroid

## Abstract

**Background:** In this study, a modified technique of resectoscopic slicing with a common bipolar loop was introduced, which facilitated the complete removal of the submucous fibroid inside the uterine cavity without any novel equipment.

**Results:** Compared with the classical technique, our modified procedure possessed a shorter operation time (22.9 ± 7.3 vs. 38.9 ± 13.0 min, *p* < 0.05) and a smaller distending media volume (1,495.6 ± 540.1 vs. 2,393.1 ± 719.4 ml, *p* < 0.01).

**Conclusion:** As a result, the current study suggested that the enucleation of submucous fibroid under hysteroscopy could be achieved by using only the bipolar loop, which reduced the consumption for novel equipment and enhanced the safety of the technique.

## Introduction

Uterine fibroids are the most common benign tumor found in the female genital tract. Localization of uterine fibroids seems to be an important factor in determining the severity of symptomatology. Indeed, submucous fibroids may induce severe clinical symptoms such as secondary anemia due to excessive bleeding. Furthermore, submucous fibroid correlates with poor reproductive outcomes for lowering the implantation rate and increasing the abortion rate (1, 2). As a result, submucous fibroid, even small in size, often causes severe clinical symptoms and influences women's quality of life.

Hysteroscopic myomectomy currently represents the standard minimally invasive surgical procedure for treating submucous fibroid (3). Over the years, many techniques for hysteroscopic myomectomy of submucous fibroid, such as cold loop technique (4, 5) and Litta's enucleation “in toto” technique (6), have been described and aimed to completely excise fibroid by one-step procedure and hence avoid both short-term and long-term complications. The “cold loop” myomectomy was first reported by Mazzon in 1995, which was characterized by a sequence of three different operating steps: (i) excision of the intracavitary component of the fibroid, (ii) enucleation of the intramural component of the fibroid using a special cold loop, and (iii) the “neoformation” is completely and safely excised using a cutting loop. The Litta's enucleation “in toto” technique is a one-step hysteroscopic myomectomy. A special thermal 90° electrode is required to open the mucosa covering the fibroid and push the fibroid into the uterine cavity, enabling the surgeon to resect the intramural component safely and completely. However, the wonderful techniques come up with some new special instruments, which definitely increase the medical expenses of patients. For example, the cold loop technique needs a series of Mazzon's mechanical loops (Karl Storz, Germany), and another Littar's enucleation technique needs Collin's electrode (Karl Storz, Germany). All the aforementioned disposable instruments cost additional money.

In our hospital, a system of operating hysteroscope with traditional bipolar loop is the only instrument used for the treatment of submucous fibroid, and the classical technique of resectoscopic slicing was previously used widely. However, this technique is limited by the use of electrosurgery during the removal of the intramural component of the fibroid, which would result in inevitable damage of the surrounding healthy myometrium, and by the multistep procedure since it cannot enucleate the fibroid prior to resection. Therefore, we made efforts to modify the classical technique, by using the common bipolar loop to enucleate the fibroid first, so as to finish the one-step excision procedure and preserve surrounding healthy myometrium.

## Materials and Methods

### Subject Recruitments

Fifty-five patients, who underwent resectoscopic myomectomy from July 2018 to December 2019 at the Shanghai Changning Maternity and Infant Health Hospital, were included in this retrospective study. The approval from the institutional review board was obtained for data collection.

Information such as age, birth history, and menstruation was registered. Blood tests for the function of hemoglobin, liver, and kidney, as well as electrocardiogram examinations, were performed before operation. Hemoglobin <110 g/L was diagnosed as anemia. Transvaginal ultrasonography was performed to assess the sizes of uterus (using the formula: length + height + width) and the submucous fibroid (using the maximum diameter).

The classification adopted by the European Society for Gynaecological Endoscopy (ESGE), which considers only the degree of myometrial penetration of the submucous fibroid, is currently used in this study as follows: G0: pedunculated fibroid, completely endocavitary and lacking in intramural extension; G1: submucous fibroid with intramural extension <50%; and G2: submucous fibroid with intramural extension higher than 50% (7).

The inclusion criteria were as follows: (1) patients with submucous fibroid diagnosed by transvaginal ultrasonography, which was <5 cm; (2) patients who were diagnosed with only G1 and G2 fibroids (under hysteroscopy); (3) patients with a uterus that is <10 weeks pregnant, or the uterine cavity depth <12 cm; (4) patients with no malignant lesions shown by the Thinprep cytology test of cervix.

The exclusion criteria were as follows: (1) patients who were contraindicated to hysteroscopic operation: (a) fever higher than 37.5°C, (b) lower genital tract infection or pelvic infection, (c) serious vaginal bleeding, (d) non-excluded cervical cancer, (e) serious medical and surgery complication; (2) patients who were pregnant or postmenopausal; (3) patients with acute infection in the reproductive tract; (4) patients who were diagnosed with G0 submucous fibroid.

### Hysteroscopic Myomectomy

Nineteen patients underwent the modified technique of resectoscopic slicing, and the other 36 patients underwent classical technique of resectoscopic slicing. Surgical procedures were performed by three expert endoscopic gynecologists with the same hysteroscopic ability and educational background. The cervix was softened by the intravaginal application of 0.6 mg misoprostol (Zizhu Pharmaceutical Ltd, Beijing, China), 4 h before operation. Under intravenous general anesthesia, all patients were placed in the lithotomy position.

Surgical procedures were performed using a 9-mm resectoscope with a 30° optical system with bipolar energy (SM10, Simai, Zhuhai, China). A traditional bipolar loop electrode is used through whole procedure.

The uterine cavity was distended by the 0.9% sodium chloride solution, constantly irrigated by an automatic fluid pump (U9522, ShenDa, Shenyang, China), with a pressure of 80–100 mmHg (1 mmHg = 0.133 kPa).

After exploring the uterine cavity, the submucous fibroid was described as follows: (1) the position of the submucous fibroid in the cavity, classified as fundus, anterior/posterior wall, and lateral wall; (2) the size of the submucous fibroid.

The following parameters relating to the operation were recorded: operation time, amount of bleeding during operation, and volume of distending fluid used during operation. Complications such as fluid overload, uterine perforation, infection, and secondary dysmenorrhea were also recorded.

### Modified Technique of Resectoscopic Slicing

**Figure 1:**
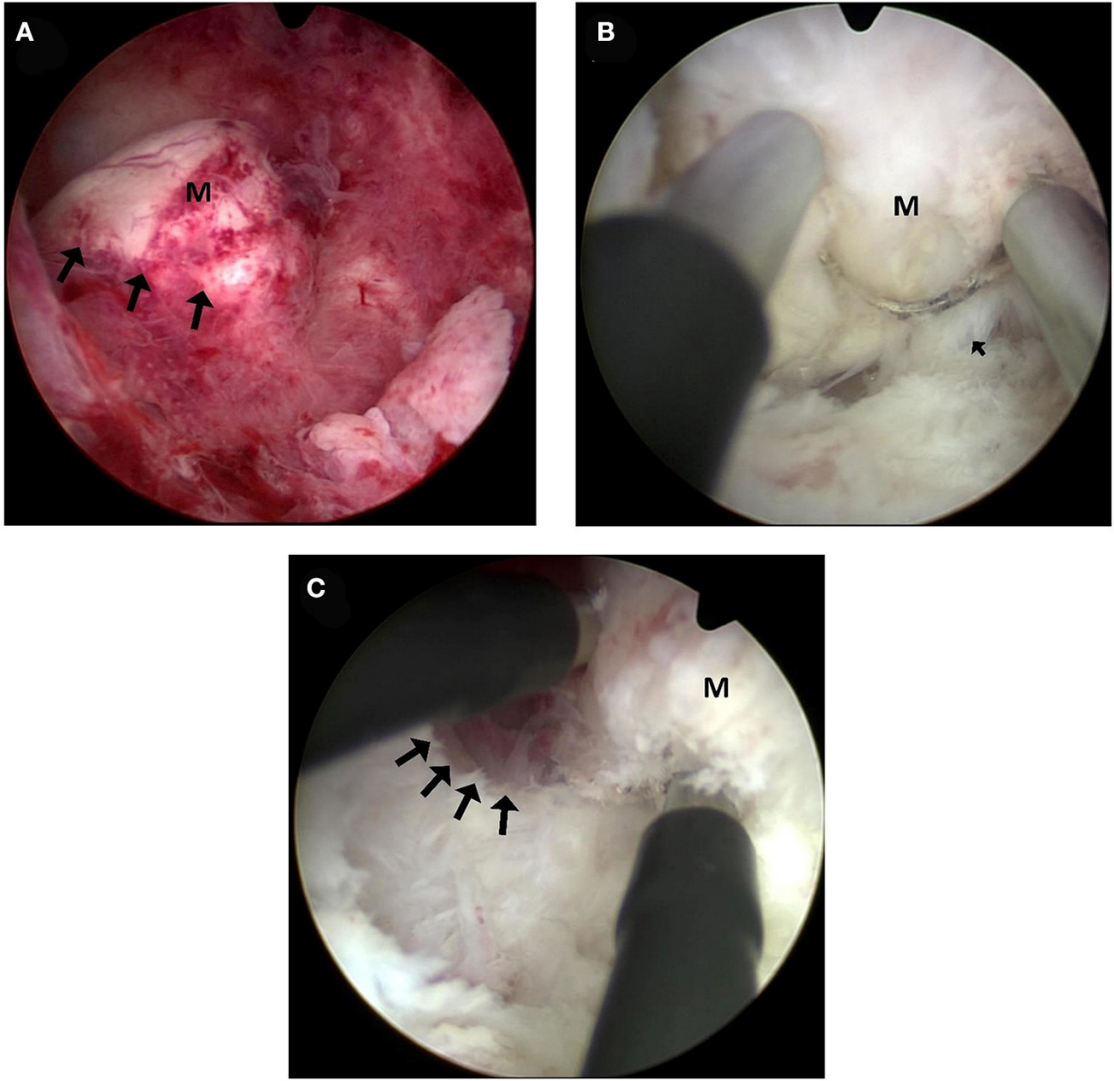
Hysteroscopic images of the modified technique. **(A)** Submucous fibroid under hysteroscopy. Arrows indicate the places where the endometrium would be cut open. M: submucous fibroid. **(B)** A space between the fibroid and the myometrium was opened. The bipolar loop with the energy of coagulation was used to lacerate the connective tissue bridges (arrows) along the surface of the fibroid. The fore-end of hysteroscope was inserted into the plane and used in a mechanical way along the surface of the fibroid. M: submucous fibroid. **(C)** Enucleation of the intramural component of the fibroid. At the end of the enucleation phase, the intramural part of the fibroid was almost dislocated into the uterine cavity. M: submucous fibroid.

After exploring the uterine cavity, the passage between the fibroid and the adjacent myometrial tissue was identified. The endometrium was scratched off next to the fibroid using bipolar loop in order to find the cleavage plane between the fibroid and the myometrium, as demonstrated in Figure 1A.

Enucleation of the intramural component of the fibroid: after the cleavage plane was identified, the bipolar loop of the resectoscope was inserted and the space between the fibroid and the myometrium was enlarged with coagulation at the energy of 100 W. Then, the fore-end of hysteroscope was used in a mechanical way along the surface of the fibroid, to progressively produce blunt dissection away from the myometrial wall. During this procedure, the bipolar loop with the energy of coagulation was used to lacerate the slender connective bridges along the surface of the fibroid, usually recognized clearly by its smooth, white, and compact appearance, as shown in Figure 1B.

Excision of the intramural component: At the end of the enucleation phase, the intramural part of the fibroid was almost dislocated from the inside of the uterine cavity, as can be seen in Figure 1C. At this point, it could be treated as a G0 fibroid with a total intracavitary development, and therefore, it was able to be excised completely and safely by means of the usual progressive excision using the same bipolar loop (the cutting energy is 120 W).

### Classical Technique of Resectoscopic Slicing

**Figure 2:**
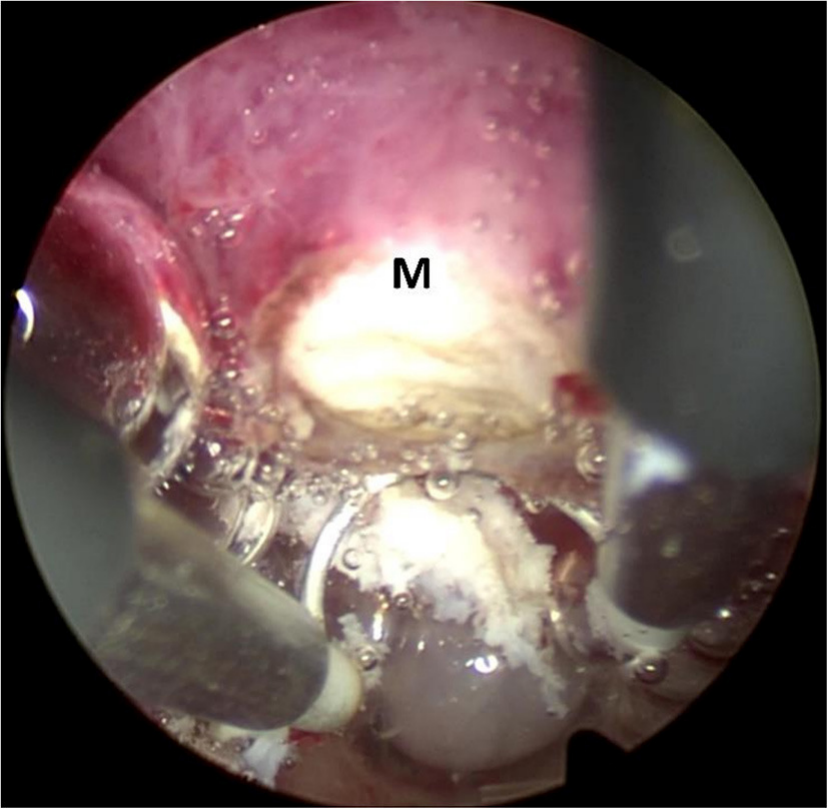
Classical technique of resectoscopic slicing. The bipolar loop was carried beyond the fibroid and begun from the top of the fibroid with the progression in a uniform way toward the base. M: submucous fibroid.

The classical resectoscopic excision of submucous fibroid was carried out with the technique of slicing. The bipolar loop was brought beyond the fibroid, and the cutting only happened during the backward movement of the loop. Excision began from the top of the fibroid and progressed in a uniform way toward the base, as seen in Figure 2. The fibroid was cut off into several smaller parts, which could be removed out of the cavity.

### Follow-Up

The first follow-up was arranged at the time of 1–3 months after hysteroscopic myomectomy. The follow-up included vaginal spotting after operation, menstruation change, and secondary dysmenorrhea. Transvaginal ultrasonography examination was undergone to assess whether any residual submucous fibroid was occurred. For patients with anemia or hypermenorrhea before operation, a blood test for hemoglobin was performed.

### Statistical Analysis

All data were analyzed using SPSS statistics 20.0 software (IBM SPSS statistics 20.0, Armonk, NY, United States). Categorical data were expressed as frequency or percentage and compared with an χ^2^ test. Continuous data were expressed as the mean ± SD and analyzed using t-test or ANOVA as appropriate. *p* < 0.05 was considered to be statistically significant.

## Results

### General Information of Patients

The average age of the patients in the modified technique group was 36.9 ± 6.6 years, and that in the classical technique group was 39.8 ± 4.4 years (*p* = 0.055). In the modified technique group, 15 cases (78.9%) were parous, 10 cases (52.6%) had a complaint of hypermenorrhea, and 10 cases (52.6%) had secondary anemia, with an average of 108.3 ± 20.6 g/L hemoglobin before operation. The uterus size was 178.1 ± 27.8 mm, and the fibroid size was 22.0 ± 7.6 mm. There was no significant difference between the two groups, as shown in Table 1.

**Table 1:** Baseline data of patients in the study.

		**Modified technique group**	**Classical technique group**	***p***
		**(*N* = 19)**	**(*N* = 36)**	
Age (years)		36.9 ± 6.6	39.8 ± 4.4	0.055
Parity (*N*, %)	Nulliparous	4 (21.1)	4 (11.1)	0.320
	Parous	15 (78.9)	32 (88.9)	
Hypermenorrhea (*N*, %)		10 (52.6)	15 (41.7)	0.437
Hemoglobin (g/L)		108.3 ± 20.6	113.5 ± 21.8	0.395
Anemia (*N*, %)		12 (63.2)	17 (47.2)	0.260
TVS (mm)	Uterine	178.1 ± 27.8	174.1 ± 26.6	0.607
	Submucous fibroid	22.0 ± 7.6	24.0 ± 12.6	0.523

*Anemia: Hemoglobin (g/L) ≤ 11 0g/L before operation*.

*TVS: Transvaginal ultrasonography was performed for the measurement of the uterus size (using the formula: length + height + width) and the size of submucous fibroid (using the maximum diameter)*.

### Perioperative Outcomes

In the modified technique group, 13 cases (68.4%) were located in the anterior/posterior wall, 4 cases (21.1%) in the lateral wall, and 2 cases (10.5%) in the fundus. No significance was observed among groups (*p* = 0.075).

The operation time was 22.9 ± 7.3 min, and the volume of distending media used during the operation was 1,495.6 ± 540.1 ml in the modified technique group. Meanwhile, the corresponding values for the classical technique group were 38.9 ± 13.0 min and 2,393.1 ± 719.4 ml. Significances were observed in both indices between two groups (*p* < 0.001), as summarized in Table 2.

**Table 2:** Perioperative data of patients in the two groups.

		**Modified technique group**	**Classical technique group**	***p***
		**(*N* = 19)**	**(*N* = 36)**	
Fibroid location (*N*, %)	Anterior/posterior	13 (68.4)	21 (58.3)	
	Fundus	2 (10.5)	8 (22.2)	0.561
	Lateral	4 (21.1)	7 (19.4)	
Fibroid classification	G1	4	7	0.887
	G2	15	29	
Operation time (min)		22.9 ± 7.3	38.9 ± 13.0	<0.001
Volume of distending media (ml)		1495.6 ± 540.1	2393.1 ± 719.4	<0.001
Bleeding (ml)		15.3 ± 7.0	19.4 ± 10.7	0.087

### Postoperative Outcomes

Table 3 shows the postoperative outcomes. In the classical technique group, fluid overload occurred in three cases (8.3%) and uterine perforation occurred in one case (2.8%). In comparison, none of the postoperative complications occurred in the modified technique group.

**Table 3:** Postoperative data of patients in the two groups.

	**Modified**	**Classical**
	**technique group**	**technique group**
	**(*N* = 19)**	**(*N* = 36)**
Fluid overload (*N*, %) ^a^	0 (0)	3 (8.3)
Uterine perforation (*N*, %) ^a^	0 (0)	1 (2.8)
Infection	0	0
Residual submucous fibroid	0	0
Hypermenorrhea correction (*N*, %)	10 (100)	15 (100)
Anemia correction (*N*, %)	12 (100)	17 (100)
Secondary dysmenorrhea/amenorrhea	0/0	0/0

a *Differences between the two groups were not statistically significant (p = 0.286)*.

According to the transvaginal ultrasonography after operation, residual submucous fibroids were not seen in any cases of the two groups. All cases complaining of hypermenorrhea (10 cases in the modified technique group and 15 cases in the classical technique group) were improved, and the secondary anemia was corrected.

## Discussion

Submucous fibroid in the uterus is a common clinical disease, accounting for 5.5–10% of uterine fibroid. In 1976, the advent of hysteroscopic myomectomy, which was first reported by Neuwirth et al. (8), radically changed the treatment of submucous fibroid. Ideally, hysteroscopic myomectomy should be a simple, well-tolerated, safe, and effective treatment, which is quite important for child-bearing women since it minimizes the impact of surgery on reproductive tract with no pelvic operation, no scar left in the uterus, and no adhesion in the pelvic cavity. It is also of great importance for infertility. Litta et al. reported that 104 infertile women with submucous fibroid who underwent the hysteroscopic myomectomy finally reached a total pregnancy rate of 85.8%, despite their fibroid classification (2). Hence, hysteroscopic myomectomy might be the optimal method to treat submucous fibroid, especially for infertility, due to the less invasive nature and better results (9).

The intramural extension of submucous fibroids, on the other hand, influences the chance of achieving complete resection in the one-step procedure, and it is sometimes associated with a higher risk of complications. Conventionally, G1 and G2 fibroids that could be removed hysteroscopically should not exceed 4 cm, even though reports mentioned about the removal of larger submucous fibroids (10). Recently, several hysteroscopic techniques have been proposed. Most of the techniques have the same objective as making the intramural component to be an intracavitary protrusion, alike the G0 fibroid actually. The “cold loop” technique and Litta's technique are widely used (11). However, these two techniques of resectoscopic myomectomy need not only a traditional cutting loop but also a special new loop, such as the Mazzon's mechanical loops for the “cold loop” technique and the Collin's electrode for the Litta's technique, either of which cost much money. Meanwhile, the surgeon has to alternate different loops in various phases during the procedure. In our study, the modified technique did not require special new resectoscopic loop. After the identification of the passage between the fibroid and the adjacent myometrial tissue, enucleation of the intramural component of the fibroid was achieved by blunt dissection using hysteroscope and coagulation energy using bipolar loop. And the same bipolar loop completed the following excision of intracavitary “neoformation.” Therefore, no alteration of the instrument was required during the procedure. Recent studies reported that any iatrogenic pseudocapsule damage may alter neurotransmitter function during successive myometrial healing, impacting negatively on uterine repair, with ultimately impaired uterine muscle function during pregnancy, labor, and delivery (12, 13). Hence, gynecologists do effort to improve the hysteroscopic myomectomy as to retain pseudocapsule. Similar to the Litta's technique and “cold loop” technique, our modified technique does good at the preservation of pseudocapsule. During the procedure in the classical resectoscopic excision of intracavitary fibroid, the fragments of resected fibroid and the accumulated gas bubbles in the cavity produced by bipolar loop with cutting energy may interfere with the clearance of the vision. Thus, the resectoscope must be taken out and in several times during the surgery for removing these obstacles. Our modified technique had a good maintenance of a clear vision, because (a) we completed the enucleation of the intramural component of the fibroid first, and decreased the fragments of resected fibroid; and (b) coagulating energy was used during enucleation, which rarely produced gas bubbles compared with cutting energy. Consequently, clear vision made the surgery safer, and probably shorter, due to the reduced amounts of fragments and gas bubbles, as well as withdrawal of the resectoscope and interruption during the operation. It is also important to note that the modified technique we introduced in the current study was never a substitution of either the Mazzon's or Litta's technique. On the contrary, we ought to provide another doable choice with less cost especially for places that are relatively short of medical resources.

Furthermore, some studies have revealed the limits of hysteroscopic myomectomy on parameters such as the size, location, and position of the fibroid, which was correlated with a variety of complications, such as infection, cervical laceration, uterine perforation, and even fluid overload (14). The serious complications of fluid overload are caused by an overabsorption of distending media and subsequent electrolyte disorder. In this study, no fluid overload in the modified technique group was observed, comparing with three cases in the classical technique group, although significant difference had not been seen possibly due to the sample size. We speculated that the reason for no fluid overload could be as follows: (1) during the enucleation of the fibroid, the application of electrocoagulation decreased the severing of submucous blood sinuses and (2) the shorter operation time decreased the use of distending media.

Apart from all the advantages, cautions should be made on the safety issues of this technique. Owing to the restricted sources, the old ESGE classification was used for the evaluation of fibroids during the study. However, STEP-W classification might be a better system for the classification of fibroids and also for further evaluation of advantages of methodology during resectoscopic slicing. In addition, during the enucleation of the fibroid, electrical coagulation was used to cut the fibroconnectival bridges, which decreased the risk of bleeding and avoided large area of rip damage, which might be processed into uterine perforation later. However, the injury caused by coagulation might result in potential complications like intrauterine synechiae, which is of great importance especially in women who were seeking for pregnancy. Although none of amenorrhea or dysmenorrhea was observed in the current study during the follow-up, the real rate of post-surgical intrauterine synechiae should be monitored in the future, and hence, a post-surgical follow-up with diagnostic hysteroscopy was suggested to be performed. Moreover, similar to the classical technique, the modified technique makes the patient at the risk of uterine perforation with thermal loop particularly in the case of G2 myomas with a thin free myometrial margin, and thus, synchronous monitoring by ultrasound is strongly suggested.

## Conclusion

The modified technique of resectoscopic slicing in the current study is a technique of enucleation using common bipolar loop, which does not need any novel instrument. It makes efforts to preserve surrounding healthy myometrium and finish the one-step excision procedure. Because this technique maintains a clearer view of the surgical field, it has a shorter operation time compared with the classical technique. However, the safety of the modified technique, such as post-surgical intrauterine synechiae rate, needs to be studied further.

## Data Availability Statement

The raw data supporting the conclusions of this article will be made available by the authors, without undue reservation.

## Ethics Statement

The studies involving human participants were reviewed and approved by Shanghai Changning Maternity and Infant Health Hospital. The patients/participants provided their written informed consent to participate in this study. Written informed consent was not obtained from the individual(s) for the publication of any potentially identifiable images or data included in this article.

## Author Contributions

WZ and JL contributed equally in drafting this work. WZ, JL, QW, YL, CW, and CM contributed to the design and analysis of the work. CM contributed to the interpretation of the data and critical analysis. All authors contributed to the article and approved the submitted version.

## Funding

This study was supported by a Fund Project of Shanghai Municipal Health Commission (201840312), China.

## Conflict of Interest

The authors declare that the research was conducted in the absence of any commercial or financial relationships that could be construed as a potential conflict of interest.

## Publisher's Note

All claims expressed in this article are solely those of the authors and do not necessarily represent those of their affiliated organizations, or those of the publisher, the editors and the reviewers. Any product that may be evaluated in this article, or claim that may be made by its manufacturer, is not guaranteed or endorsed by the publisher.
